# Mental health policy in Kenya -an integrated approach to scaling up equitable care for poor populations

**DOI:** 10.1186/1752-4458-4-19

**Published:** 2010-06-28

**Authors:** David Kiima, Rachel Jenkins

**Affiliations:** 1 Mental Health Directorate, Ministry of Health, Nairobi, Kenya; 2 Department of Health Services and Population Research Department, Institute of Psychiatry, Kings College London, de Crespigny Park, London, SE5 8AF, UK

## Abstract

**Background:**

Although most donor and development agency attention is focussed on communicable diseases in Kenya, the importance of non-communicable diseases including mental health and mental illness is increasingly apparent, both in their own right and because of their influence on health, education and social goals. Mental illness is common but the specialist service is extremely sparse and primary care is struggling to cope with major health demands. Non health sectors e.g. education, prisons, police, community development, gender and children, regional administration and local government have significant concerns about mental health, but general health programmes have been surprisingly slow to appreciate the significance of mental health for physical health targets. Despite a people centred post colonial health delivery system, poverty and global social changes have seriously undermined equity. This project sought to meet these challenges, aiming to introduce sustainable mental health policy and implementation across the country, within the context of extremely scarce resources.

**Methods:**

A multi-faceted and comprehensive programme which combined situation appraisal to inform planning, sustained intersectoral policy dialogue at national and regional level; establishment of a health sector system for coordination, supervision and training of at each level (national, regional, district and primary care); development workshops; production of toolkits, development of guidelines and standards; encouragement of intersectoral liaison at national, regional, district and local levels; public education; and integration of mental health into health management systems.

**Results:**

The programme has achieved detailed situation appraisal, epidemiological needs assessment, inclusion of mental health into the health sector reform plans, and into the National Package of Essential Health Interventions, annual operational plans, mental health policy guidelines to accompany the general health policy, tobacco legislation, adaptation of the WHO primary care guidelines for Kenya, primary care training, construction of a quality system of roles and responsibilities, availability of medicines at primary care level, some strengthening of intersectoral liaison with police, prisons and schools, and public education about mental health.

**Conclusions:**

The project has demonstrated the importance of using a multi-faceted and comprehensive programme to promote sustainable system change, key elements of which include a focus on the use of rapid appropriate assessment and treatment at primary care level, strengthening the referral system, interministerial and intersectoral liaison, rehabilitation, social inclusion, promotion and advocacy to mobilize community engagement.

## Background

Poverty is a major problem in sub-Saharan Africa and while other regions are achieving significant economic growth, the number of people living in poverty in Africa has nearly doubled to 314 million in the last 20 years, despite some African countries achieving economic growth averaging 5.1% in recent years [1]. Common problems of recurrent drought, famine, anarchy, poor educational development, internal and across border migration, HIV/AIDS, and intrastate conflict are now major threats to economic development in Sub Sahara Africa., and Kenya is no exception, with regular famine in some areas, political and economic conflict, and border troubles. National poverty levels had declined between 1997 and 2006 from 52.3% to 43.9%, but the recent conflict in 2008 damaged the tourist industry, and has greatly aggravated economic problems and poverty, while climate change is affecting rainfall, with ensuing famine in various parts of the country.

Mental disorders have only attracted limited attention in global efforts at alleviating poverty through investment in health, despite evidence of their impact [2]. Substantial and enduring improvements in mental health services require an integrated policy and strategy, including systematic educational interventions to equip service providers with necessary knowledge and skills, public education to raise awareness of the importance of mental health and mental disorders, combined with organisational reforms to enable interventions to be embedded in the health system and in routine care [3,4]. Unlike the large scale investments in vertical communicable disease programmes, there have only been very limited investments in mental health, mental disorders and other non-communicable diseases. The attention to vertical programme delivery, and use of non-public health sector actors and infrastructure has diverted attention and funds away from basic strengthening of public health care systems in Africa, so that despite considerable investment, there are still only 1 or 2 nurses and clinical officers for 10,000 population in SSA. Where available for mental health, donor funding has usually been short-term, uni-sectoral, and focussed on vertically implemented projects, with inadequate attention to sustainability [5].

This paper describes an integrated approach to mental health policy development and implementation in Kenya within two main phases of work, structured as two consecutive funded projects, the first a wider policy development project for both Kenya and Tanzania, funded by the UK Department for International Development (DFID) between 2001 and 2004, and carried out by the WHO Collaborating Centre, Institute of Psychiatry, London, in partnership with the Ministry of Health in Kenya and the Ministry of Health and Social Welfare in Tanzania; and the second a programme of training and supervision for primary care, funded by Nuffield 2005-2010, and carried out by a partnership between the Ministry of Health, Kenya Medical Training College, Kenya Psychiatric Association and the WHO Collaborating Centre, Institute of Psychiatry [6,7].

## Methods

### Overall approach

The Kenya mental health programme of work was multi-faceted and comprehensive, and was conducted in liaison with the Kenya Ministry of Health, DFID UK and Kenya offices, and the WHO Kenya office. The first phase of work 2000-4, funded by DFID, undertook detailed situation appraisal of mental health within Kenya; developed locally tailored and integrated mental health policy and a strategic action plan; developed mechanisms for sustainable implementation of policy across the country, using locally available resources and integrated into local systems; and evaluated progress to fine tune implementation. The second phase of work 2005-10, funded by Nuffield, aimed to integrate mental health into primary care by giving 3000 health workers exposure to mental health training, combined with supervision, guidelines, medication supply and data collection. Policy development and implementation also continued during this second phase.

### Country situation appraisal

Situation appraisal was conducted firstly by identification and analysis of national and local data, and ministry and other documents; secondly by site visits to relevant sectors (health, education, social welfare, police, prisons, NGOs) at national, regional, district and primary care levels, accompanied by detailed consultation and discussion with professionals, clients, families and other stakeholders; thirdly by stakeholder workshops spearheaded by the Ministry of Health; fourthly using the information collected above to construct a mental health country profile of Kenya - a structured systematic assessment of context, needs, resources, provision and outcomes (aligned to the model developed by Murray and Frenk [8], containing qualitative and quantitative data, and compiled by multiple stakeholders [9-11]; fifthly by conducting a small pilot epidemiological survey of a 1 in 50 sample of a 50,000 population surveillance site which will be reported elsewhere, sixthly by conducting a small survey of knowledge and attitudes to mental disorder in primary care[12]; and lastly by a focus group with traditional health practitioners [13]

### National policy dialogue

The authors undertook sustained policy dialogue on mental health with the Ministry of Health, Ministry of Social Welfare, Ministry of Education, Police, Prisons, Child Protection etc. about both generic and specific issues. The generic issues included firstly the need for national mental health policy; secondly the need for a strengthened mental health section within the Ministry, in order both to represent mental health in generic policy development and to coordinate mental health for Kenya; and thirdly the need to integrate mental health into generic health sector reforms.

The specific policy dialogue comprised consideration of the needs, strengths and challenges identified in the situation appraisal. This guided the development of written policy and strategic action plans to address Kenya's needs in the light of Kenya's context and resources, culminating in the preparation of Mental Health Policy Guidelines to support the overall Kenyan Health Policy. In addition and very crucially, mental health was integrated into: firstly, the health sector reform plans and general health policy; secondly, the Kenya Essential Package of Health Interventions so that mental health appears as an integral component of health care at all levels, with defined interventions from primary care to tertiary hospitals. Consequently, the National Essential Drugs List outlines the types of psychotropic medicines to be provided from tertiary to primary care treatment settings. The national Health Management Information System is still under development, but intends to include mental health in more detail than hitherto[14].

### Organisational and operational interventions

Organisational and operational interventions, designed in collaboration with the Ministry of Health and iteratively modified during stakeholder consultation workshops, included expansion of the Directorate of Mental Health, within the Division of Curative Services; construction of guidelines for roles and responsibilities on mental health within the various tiers of the overall health service together with delineation of the potential contributions of other key sectors; capacity building workshops both to establish and strengthen district mental health coordination and supervision of mental health services in primary care, and to strengthen district capacity to ensure the inclusion of mental health in district annual operational planning; coordination and supervision of primary care by district mental health nurses and district public health nurses, adaptation of the WHO primary care guidelines for mental health for Kenya; and establishment of systems for continuing professional development for primary care [6,7].

## Results

### Situation Appraisal

Additional file 1 summarises the key integrated findings of the situation appraisal, derived from the documentary analysis, the site visits and consultations with the various sectors and various tiers of the health service, the regional workshops, and the construction of the mental health country profile for Kenya [9,11].

#### The context

Kenya is one of the poorest countries in the world. The population is 34M and life expectancy is 54 years. More than 1 in 10 children die before the age of 5, and 4 women out of every 1,000 die in child birth. 9% women and 5% of men have HIV [15], with rates of 10% or higher in urban areas and 6% in rural areas. Inequality is high, and Gross National Income per capita in 2006 was 580 USD, lower than the SSA average 842 USD. In 2008 there was widespread violence and ethnic division following a disputed general election in the context of unemployment, economic disparities and widespread concerns about access to ancestral lands. This has been compounded by, worsening border troubles with Sudan and Somalia. Last year's conflict resulted in damage to physical assets, displacement of about 300,000 people (about 1 percent of the population); i) the loss of confidence among investors and tourists; and damage to social capital. Around 1000 internally displaced people remain in scattered camps despite government efforts at resettlement. Annual inflation in early 2009 had risen to 29%, and so while national absolute poverty had declined form 52.3% in 1997 to 45.9% in 2005/6, since the conflict the World Bank has estimated that the poverty headcount has increased by 22 percent and a measure of severe poverty has gone up by 38 percent which means gains made over the past five years on this front have been reversed [16].

#### Health service configuration

The health service is broadly structured into six levels; the national referral hospitals (level 6), provincial general hospitals, (level 5), district general hospitals (level 4), health centres (level 3), dispensaries (level 2) and volunteer community health workers (level 1).

In 2008, as part of the establishment of the new coalition government, with a consequent doubling in the number of ministers, the Ministry of Health split into two ministries, the Ministry of Medical Services which is responsible for health delivery at national, provincial and district level, and the ministry of public health and sanitation which is responsible for health delivery in health centres, dispensaries and the community. These changes have resulted in duplication of administrative posts at provincial and district levels, and confusion amongst staff as to reporting lines, accountability and planning routes. In addition, until recently there were 72 districts, with an average catchment population 500,000; but these have recently been reconfigured first to 149, with an average catchment population of around 250,000, and now to around 250, with a catchment population of around 150,000. The required expansion of staff numbers is greatly straining a public system where there is insufficient trained human resource.

#### Financing, resource allocation and provider payment system

Mental health care in Kenya is predominantly government funded. Budgets, originally centralised in the MOH, were decentralised in 2008 to local district councils as part of Treasury reforms, leaving the MOH with its core technical functions of policy formulation, legislation formulation, standards and guidelines for service delivery, regulation, strategic planning coordination, performance monitoring and evaluation, funds sourcing and mobilisation. All the health facilities have been gazetted as audit units under the Exchequer and Audit Act, empowering them to receive funds directly from the exchequer, treasury and account for them. Level 2-6 are now receiving funds, sent electronically from Treasury, channelled through the MOH, to the facilities. Districts have therefore lost their earlier role of distributing funds to health facilities, but they have their own funds raised from cost sharing revenues for running various activities (Clients are asked to pay a small fee for each consultation. This fee goes to the cost sharing fund of each facility where it is used for service developments). Considerable capacity building is required and is underway to ensure accountability, transparency and proper utilisation of public funds.

Funds for mental health remain extremely limited, and strategic advocacy is required for adequate prioritisation of mental health in each district and province, and this skill was therefore now included in the CPD of district and provincial mental health in charges run by this project (see below).

#### Mental health needs

Hitherto, there had been no mental health epidemiological studies at household level in Kenya. This project conducted such a study of Maseno district, near Kisumu, a rural population of 50,000 people, and drew a 1 in 50 sample based on the earlier census which had enumerated all households. The results will be reported elsewhere.

#### Specialist outpatient and inpatient services

Evaluation of the status of mental health services in the country by the Ministry of Health in collaboration with this project reconfirmed that the country's health care system operates under extremely resource-restricted conditions, in terms of infrastructure, manpower and finances. Mental health specialist care is largely delivered at district level by psychiatric nurses running outpatient clinics, by psychiatric nurses at provincial levels running inpatient units and outpatient clinics, and by the national referral hospitals at Mathari, University of Nairobi, Gil Gil hospital and Moi University.

The total number of hospital beds for a population of over 38 million is 1114 (750 beds but around 500-600 occupancy at Mathari; 40 beds at Moi University teaching and referral hospital at Eldoret; 100 beds at Gil Gill (established for long stay patients from Mathari but now takes acute cases as well); 6 provincial units of 22 beds each at Nakuru (Rift Valley), Kisumu (Nyanza), Nyere (Central), Embu (Eastern), Port Reitz (Coast) Kakamega (Western); and 5 district units (Machakos 22, Isiolo 10, Kerugoya 20, Muranga, 20, Meru 12, Siaya, 8,) which works out at less than 1 bed per 34,000 population. In practice, in most provinces there are only 22 beds per 4 M i.e. 1 bed per 200,000 population. With the prevalence of probable psychosis running at over 1%, it would be helpful for every district hospital to have a 20 bed inpatient unit for brief admissions to assess and stabilise complex cases. This would still leave more than 99% of people with psychosis to be managed in the community by the health centre and dispensary levels.

Kenya has its own self sustaining training programme for psychiatrists at the University of Nairobi, producing around 6 new psychiatrists per year, and the numbers have expanded from 16 psychiatrists in the public service in 2001, to 46 in 2009. In addition, there are 24 psychiatrists working in private practice In Kenya and another 20 outside the country. A further five trained in Kenya have already died. The psychiatrists in the public service are deployed to the national hospital Mathari (4 plus 1 on long term sick leave), the MOH HQ (3 plus 1 on secondment to the WHO country office plus 1 provincial director of medical services in Nairobi), the University of Nairobi (10), Kenyatta Hospital (6), Kenyatta University (2), Armed forces hospital (1), Moi University (6), Provincial hospitals (6 -Garissa has none), plus 5 placed in the district hospitals of Machakos (1), Thika (1), Muranga (1), Meru (1), and Kisii (1).

Thus it can be seen that the majority of the psychiatrists are in Nairobi, and that the effective psychiatrist-population ratio outside Nairobi is 1 psychiatrist per province of 3-5 million people. North Eastern Province, an extremely challenging environment adjoining Somalia, currently has no psychiatrist or psychiatric nurse. At the current rate of production it will take about 100 years to produce enough psychiatrists to have one in each district, taking account of retirement, and assuming no further brain drain. The University of Nairobi has also started training clinical psychologists since 2000 (currently 37 students) and psychiatric social workers since 2005 (currently 1 student). There is also a new post graduate diploma in substance abuse with 2 students.

There are 418 trained psychiatric nurses in Kenya of whom only 250 are currently deployed in psychiatry (the other 250 are deployed in general medical, surgical and obstetric services or in HIV centres), 70 are in Mathari National Hospital, leaving 180 in the districts and provinces, resulting in only less than 1 psychiatric nurse per new district or 2-3 psychiatric nurses per old district. Many psychiatric nurses have retired, died, left the country or work in NGOs, especially linked to HIV activities, and new applicants for mental health nurse training are dwindling. Thus 2009 will see the production of only 1 new psychiatric nurse for Kenya. There is 1 medical social worker in each province but none at district level, and there are social workers in prisons, probation services, the children's dept and the ministry of Social services. There are a handful of psychologists in university or private practice in Nairobi.

Thus the specialist service for nearly all regions and districts is largely delivered by extremely overstretched mental health nurses, who have had no access to continuing professional development throughout their careers until that afforded by phase 2 of this programme of work, funded by Nuffield. This lack of human resource and the continued limited funding of mental health services both severely curtail access to specialist care, and this situation will rapidly get worse unless urgent action is taken to train more psychiatric nurses. The Ministry of Health is planning to offer 10 bursaries for training mental health nurses next year, but if the numbers are to expand rather than simply replace losses, that figure will need to double. The production of other specialist cadres would also benefit from support.

#### Primary care service

Primary care non-specialist services are delivered through primary care health centres (level 3) (average catchment population 10,000) and dispensaries (level 2) which are staffed by general nurses and clinical officers who have received a small amount of basic training about mental health but have not until now received detailed training in multiaxial assessment, diagnosis and treatment, and have not hitherto received any in-service training or supervision for mental health.

Primary care staff do see, diagnose and treat people with psychosis, which is generally relatively visible even to lay people. Where transport and facilities are available, they may refer very complex cases of psychosis to the district or regional level (See above). Families also often self refer directly to district, regional and even national hospitals. However, the more common mental disorders of depression and anxiety are rarely diagnosed, and even when diagnosed, are rarely treated appropriately (see below). Community oriented treatment is starting to be carried out both by PHC staff and district staff doing outreach, in liaison with families and community leaders, with gradual increasing involvement of volunteer community health workers (see below).

#### Medication

The situation appraisal in 2001/2 showed that the supply of essential psychotropic drugs did not meet demand in the country. The shortage of drugs in health care facilities forced patients and their families to either finance their own supplies, or go without them. Kenya Medical Supplies Agency KEMSA is the procurement and distribution agency for pharmaceutical and non-pharmaceutical items for minister of health facilities. Historically, it has supplied the dispensaries and health centres with drug kits according to the essential medicine list (the "push" system), but the psychotropic drugs supplied have not been adequate in numbers and variety. Until 2007, it was only hospitals at levels 4 and 5 who have been supplied with antidepressants, while all levels including health centres and dispensaries have been supplied with chlorpromazine, diazepam and phenobarbitone. Policy dialogue has now resolved this situation, with antidepressants now made available at the primary care level in the essential medicine list since 2008. The country is now moving to the "pull" system of distribution of medicines, whereby each health facility prioritises and orders its own drugs from KEMSA according to the essential medicine list. Meanwhile there are several districts where psychotropic drugs are supplied to levels 2 and 3 from the district hospital, where the district mental health in charge liaises with the district pharmacy for the level 2 and 3 to order according to their workloads and needs. The historical lack of antidepressants at health centre level has resulted in the widespread but erroneous prescription of diazepam, when in fact it is not curative for psychological problems, is disinhibiting, and is also highly addictive.

#### Non governmental organisations

There is a growing civil society interest in mental health, although still predominantly in Nairobi. The Schizophrenia Fellowship of Kenya is affiliated to the World Federation for Schizophrenia and Allied Disorders. There are also NGOs for people with autism and learning disabilities The Kenya Association of Mental Health is inoperative. Basic Needs (a UK based NGO) had a pilot mental health project in a slum area in Nairobi, and is now establishing two pilot projects in two districts in Rift valley province. There are also a number of general NGOs who are starting to take an interest in mental health, such as AMREF and FIDA. AMREF is an active NGO in Kenya which through their learning dept has been conducting upgrading distance learning for enrolled nurses to become registered nurses -this has been a national programme to upgrade all certificate holders to diploma holders in nursing, and AMREF have now approached this project to explore the possibilities of collaboration to conduct e-learning on mental health for community and hospital health workers.

The recent conflict led a number of NGOs to gain funding for short term psychosocial work, and there is growing concern the funds were spent on largely unregulated counsellors, who were not adequately integrated with health and social services to enable appropriate referrals of complex cases and who were not working to internationally accepted evidence based guidelines.

### National policy dialogue and organisational and operational interventions

#### Policy

Draft policy and strategic action plans were prepared in 2003/4, based on the detailed situation appraisal 2001-4, to address Kenya's needs in the light of Kenya's context and resources, and were taken through a lengthy process of stakeholder consultations 2004-8, led by the Ministry of Health; have been discussed in each of the CPD trainings for specialist staff and for primary care health workers; and now culminating in the preparation of Mental Health Policy Guidelines 2009 to support the overall Kenyan Health Policy. In addition and very crucially, mental health was integrated into: firstly, the health sector reform plans and general health policy 2004-9; secondly, the Kenya Essential Package of Health 2006 so that mental health appears as an integral component of health care at all levels, with defined interventions from primary care to tertiary hospitals. Consequently, the National Essential Drugs List 2007 outlines the types of psychotropic medicines to be provided from tertiary to primary care treatment settings. The national Health Management Information System is still under development, but intends to include mental health in more detail than hitherto [14]. The core components of the mental health action plan are summarised in Additional File 2.

#### Legislation

Mental health legislation is currently being revised, and a draft Code of Practice to implement the legislation has been prepared and is out for consultation before finalisation, in the context of efforts to harmonise Kenya's public health laws. The existing mental health legislation included the formation of a National Board Kenya Board of Mental Health, and it is intended to reactivate this to oversee the implementation of the new legislation and of the national policy, and it will coordinate intersectoral liaison on mental health at national level. The Board is intended to meet regularly but there have been no finances to support this. Various strategic programmes are now underway to prepare Kenya for its human resource requirements [16], to realign the national hospital at Mathari (which is run down with condemned buildings, low morale and shortage of health staff) as a principal training institution for nurses and clinical officers, and to continue the CPD programme for primary care in a sustainable way.

#### Human Resource Strategy

In the reorganised health sector, as the MOH split into two, HR strategy is key to ensuring that health service delivery is sustained. Decentralisation is the key theme in the new Ministry of Medical Services strategic plan 2008/9-2012/13, and therefore careful human resource mapping and situation analysis for HR needs is underway, to underpin human resource development. The existing acute shortage of mental health workers has been made worse by the reorganisation in the health sector, which has created the need for more than double the number of administrative posts. Due to the long term moratorium on employment imposed by the World Bank, a lot of experienced mental health workers have reached the mandatory retirement age and are now retiring in large numbers without replacement -this has made the situation more acute. Brain drain is an additional problem [17]. The agreed mental health roles and responsibilities at each level in the health system are set out in Additional file 3.

Psychiatrists have been trained in the Department of Psychiatry at the University of Nairobi, since 1982. The training takes 3 years, on top of the six year training for the basic medical degree, and the candidates are awarded a MMed in Psychiatry. The psychiatric nurses are trained in a two year post basic diploma in psychiatry, on top of a three or four year basic nurse training, which is carried out by in the Kenya Medical Training College in Mathari School of Psychiatry training MTC.

Dialogue was undertaken with the various training organisations on strengthening curricula and methods to meet Kenyan needs. The content of specialist training should reflect the wide role which professionals, who may be responsible for catchment populations ranging from 500,000 to 6M, need to play if they are to have a sustained impact on their population. Such specialist staff cannot hope to single-handedly meet such population needs, and a significant proportion of their time will need to be spent, not in direct clinical work but rather in teaching and supporting front line primary care staff to assess, diagnose and manage mental disorders, advocating for and facilitating service development, intersectoral liaison at provincial and district levels, and contributing to the annual planning process. They will need leadership, educational, mentoring/supervisory and intersectoral skills as well as clinical and research skills. Specialists also need to be able to cover the various subspecialty issues of children, adolescents, older people, forensic, rehabilitation, and liaison as there isn't enough human resource for the subspeciality division of labour that now happens in western countries. Therefore a leadership component has now been included in the psychiatry training, and a number of developed country subspecialists have been encouraged and supported to contribute to the training programme over the last few years.

As well as basic and postbasic training, continuing professional development (CPD) for all levels is crucial as staff may be in post for 30 to 40 years. Therefore the second phase of the project included provision of CPD for primary care, specialist care and public health. 1677 primary care workers (levels 2 and 3) have been trained between 2005 and 2009 [6]. 133 senior district psychiatric nurses (Level 4) and 10 provincial psychiatrists (level 5) were called up by the Ministry of Health for a one week workshop between 2006 and 2009, funded by the Nuffield project, to strengthen capacity for the skills described above. In addition 52 district public health nurses have also been trained in mental health aspects of supervision on mental health in primary care. Contemporary texts on mental health primary care, specialist care and policy, were also provided to the respective training institutions to assist with basic and postbasic training materials.

The project CPD training programme has also paid attention to the community health workers (CHWs) linked to health centres and dispensaries, who are intended by the Ministry of Health to form a bridge between the community and health workers. There is supposed to be 1 CHW per 100 households, and they are intended to be supervised by nominated health workers from level 2 and 3 (henceforward known as community health education workers (CHEWs). CHWs were formerly volunteers, but now the government is encouraging their remuneration by the community.

Our CPD training programme on mental health for primary care staff included a module on how to deliver brief mental health training to their CHWs as part of their normal regular weekly education sessions for CHWs attached to their clinics. The roll out of this has recently received a major boost by the Ministry of Public Health and Sanitation which has introduced a funded programme for districts to train the CHWs and expand their role. Many of those district staff engaged in training CHWs have attended our mental health CPD programme and are therefore incorporating those materials into the CHW training.

#### Intersectoral liaison

Policy linkages were established with other relevant ministries including the government departments responsible for police, prisons, schools, child protection, and social welfare. All have major interests in contributing to mental health policy, and have all participated in the stakeholder policy development workshops. They are each represented on the Kenya Board of Mental Health. Dedicated training courses on mental health have been run for prison nurses, and a number of prison nurses have also attended the primary care training courses.

#### Advocacy and public education

Kenya has participated in the celebration of World Mental Health day annually since inception in 1992. The national event is held on a rotational basis in each province and the minister for health presides over the national event. The national celebration is a culmination of a week long mental health activities carried out throughout the country at district levels. The minister gives a trophy and a certificate to the national winner of the national competition for the best mental health worker of the year. The climax is marked by the minister's speech which articulates the mental health issues contained in the theme for each year, accompanied by extensive media coverage. Mental health workers from different levels in the service also contribute to media programmes about mental health. They give lectures, symposia and workshops to create public awareness as well as orientation of different groups and cadres on mental health. Psychiatric hospitals and wards have open days.

## Discussion

Although most donor and development agency attention is focussed on communicable diseases in Kenya, the importance of non-communicable diseases including mental health is increasingly apparent, both in its own right and because of its influence on health, education and social goals. Mental illness is common but the specialist service is sparse and primary care is struggling to cope with major health demands. Non health sectors have significant concerns about mental health, but other health programmes have been slow to appreciate the significance of mental health for physical health targets.

The ten year programme of work described in this paper has achieved detailed situation appraisal, epidemiological needs assessment, inclusion of mental health into the health sector reform plan, essential package of health interventions, mental health policy guidelines to accompany the general health policy, adaptation of the WHO primary care guidelines, primary care training, construction of a quality system of roles and responsibilities, availability of medicines at primary care level, intersectoral liaison with police, prisons and schools, public education about mental health, and a research programme to inform future developments. In many respects this is remarkable given the small size of the project funding, but it is also a key strength in that low income countries experience many donor funded projects which work outside rather than within the system and which collapse when funding is discontinued. This ten year programme has worked closely with the Ministry of heath and other relevant ministries throughout, and has sought to integrate its work systematically into the Kenyan system, so that the system can continue to function irrespective of donor funding or of personalities.

The limitations of the programme have included limited financing, frequent changes in senior ministry personnel, and changes in ministry structures. However, despite the shortage of resources, and the international and national focus on communicable disease, mental health is in the health sector strategic plan, a sustainable programme of implementation has been achieved despite enormous constraints, and other sectors are very keen to integrate mental health into their work.

The strengths of this programme, which may be useful in planning similar policy projects elsewhere included an integrated and coordinated set of activities at multiple levels and across sectors; planning for the sustainability right from the beginning of the project; intensive policy dialogue throughout the project; and evaluation to assess impact. All training materials were widely disseminated to the workforce, local academic centres, and incorporated into curricula. Critically, the project did not develop externally funded services in danger of collapse when funding ended but rather facilitated local service development using regular ministry budgets. Although the DFID project officially lasted only three years, the partnership between the MOH and the WHOCC has continued for ten years, through the joint work on the Nuffield funded integration of mental health into primary care project, and this long term dialogue and joint working has been useful, achieving more sustainable impacts than might have been achievable in a shorter time period.

## Conclusions

The project has demonstrated the importance of using a multi-faceted and comprehensive programme to promote sustainable system change. Key elements include a focus on the use of rapid appropriate treatment at primary care level, strengthening the referral system, encouraging intersectoral liaison, rehabilitation, social inclusion and public education. Kenya has declared its commitment to mental health and this project has provided a secure and sustainable policy foundation for sustainable implementation which has developed momentum over the last several years, despite the poverty, human resource constraints, and the turbulence of the 2008 post election violence.

## Competing interests

During this period, DK was Director of Mental Health, Ministry of Health, Kenya and RJ was Director of the WHO Collaborating Centre, IOP, and in receipt of two consecutive grants, one from DFID to give mental health policy support to Kenya, and the other from Nuffield to integrate mental health into primary care in Kenya, both in partnership with the Kenya MOH.

## Authors' contributions

RJ wrote the first draft of this paper and DK and RK both contributed to subsequent drafts. DK led mental health policy in Kenya over the time period covered by this paper, supported by RJ. All authors read and approved the final manuscript.

## Supplementary Material

Additional file 1 Kenya situation appraisal 2001 Click here for file

Additional file 2 Kenya Mental Health Strategic Action Plan 2004 Click here for file

Additional file 3 **Kenya progress assessed against policy frameworks set out in Jenkins et al 2002 (final revision Feb 2001 and given on request to editor in chief of World Health Report in March 2001), World Health Report October 2001, WHO Policy Package 2003**. Click here for file
