# Supplementary material for: Mental health policy in Kenya -an integrated approach to scaling up equitable care for poor populations
Source: Int J Ment Health Syst. 2010 Jun 28;4:19. doi: 10.1186/1752-4458-4-19 (PMC2907308; doi:10.1186/1752-4458-4-19)
Supplement: Additional file 1 — Kenya situation appraisal 2001 [file 1752-4458-4-19-S1.DOC]

**Additional file 1 - Kenya situation appraisal 2001**

.

| **Policy Component** | **Kenya Situation** |
| --- | --- |
| 1. **Position of**   **Mental Health in Ministry** |  |
| ***Director of Mental Health*** | MOH has part time psychiatrist, who is based at Mathari Hospital where he is also Director of the hospital. Thus his time for policy work is limited. |
| ***Board of Mental Health*** | Infrequent and meetings of Kenya Board of Mental Health due to inadequate budgetary allocation. |
|  | ineffective meetings of the Board |
|  | lack of strategic agenda for Board |
| ***Mental health policy*** | No Mental Health Policy and Strategic Plan |
| ***linkage to PRSP and economic recovery plan*** | mental health not yet included in this process |
| ***linkages to broad health and social policy*** | too low a political profile so mental health has been excluded from relevant policies, which are hindered by its exclusion |
| ***Support to Director of Mental Health*** | Inadequate staffing of Division of Mental Health. |
| ***Prioritisation of mental health*** | Mental health is yet to be accepted as priority |
| ***representation in donor meetings by Division of Mental Health*** | Mental health not represented in donor meetings, and this becomes a vicious circle of lack of inclusion, forcing mental health to remain a partial vertical programme despite its best efforts to integrate with general health sector activities |
| ***Collaboration with Directorate of Preventive and Promotive Health services*** | very little collaboration as yet |
| ***Collaboration with Directorate of Policy and Planning*** | very little input in mental health in the development of policy and planning |
| ***Collaboration with Human Resources*** | inadequate human resource development in mental health |
| ***mental health awareness of senior MOH officials*** | awareness could be substantially improved |
| ***links with other key ministries eg criminal justice, home affairs, social welfare, education, labour.*** | links not yet well established |
| 1. **Primary**   **Health Care** | Mental health is not included in PHC work in most of the districts in spite of being an essential element of PHC as adopted by Kenya in 1982. Major lack of training guidelines, transport, information system and essential medians. |
| ***Continuing education (in- service training)*** | No continuing education in mental health for PHC staff. |
| ***Role of village health workers*** | Village health workers operative in some areas but not others |
|  | No continuing education in mental health for village health workers |
| ***Dialogue with traditional healers*** | No routine dialogue between PHC and local traditional healers about mental illness although many people with mental illness are consulting traditional healers. |
| ***Guidelines*** | No good practice guidelines or indeed any text. |
| ***PHC health information*** | No good system or recording basic categories of mental disorder |
| ***essential medicines kit*** | Problem with essential medicine kit. Volume not enough. Too short expiry dates. Not enough amitryptyline |
| ***transport for outreach*** | No transport available for staff to do out reach to people with psychosis.  . |
| ***access to rehabilitation at PHC level*** | No access to rehabilitation at community/PHC level, despite actuarial need |
| ***stigma at PHC level*** | Stigma about mental illness at community level a key problem |
|  |  |
| **Links between**  **primary and**  **secondary care** | Weak linkages in referral systems upwards and downwards. Lack of supervision and support down the system and lack of communication. |
| ***referral pathways*** | weak referral system |
| ***standards*** | no guide to expectations at each level |
| ***transport for districts*** | lack of access to transport for districts to supervise PHC is a major problem |
| ***communication*** | no resource for regular communication |
| ***regular supervision of PHCs by districts*** | No systematic supervision of PHC by districts |
| ***training for districts in how to supervise and support PHCs*** | No training for districts in how to supervise and support PHCs on mental health |
| **4. Secondary care** |  |
| ***District, provincial and zonal and national tiers*** | Tiers are not working: the tertiary role of provincial hospitals is weak or non-existent. In fact provincial hospitals operate as local district hospitals. Since there are in any case no psychiatrists, by and large, in the provincial hospitals, the provincial hospitals do not have any extra expertise, which the district hospitals don’t already have, so there is currently no added value to referring up the system.  Inadequate human resource – see below |
| ***Inpatient units*** | Insufficient coverage – some provincial hospitals have inpatient units but most districts still do not have psychiatric inpatient units (55 out of 70 district hospitals do not have psychiatric inpatient units.) |
| ***linkages between mental health service and physical health service*** | frequently relationships not good. |
| ***outpatient clinics*** | Many districts don’t have OPD for mental health |
| ***Community outreach services*** | A few districts are setting up good community services, others have not begun to think beyond inpatient beds and outpatient clinics. |
| ***Availability of medicines*** | Insufficient medicine. |
| ***Good practice guidelines for secondary care*** | No good practice guidelines for assessment, diagnosis of management (care planning) and criteria for referral to provinces or national hospital. |
| ***Rehabilitation activities on inpatient wards*** | ward activities very limited or non existent |
| ***Rehabilitation facilities at district level*** | Insufficient rehabilitation. Limited rehabilitation capacity at district level |
| ***availability of psychological treatments in secondary care*** | No current training in skills for community working, family work or specific psychotherapy and psychological skills. |
| ***Delivery of support and supervision to PHC*** | Little or no support or supervision given by district teams to primary care. |
| ***length of admissions*** | District units have a few long stay patients who they are not actively rehabilitating. National hospitals have many. People waiting far too long for proper review. Insufficient care planning. |
| ***national hospitals*** | National hospitals heavily stigmatised. Forensic cases often lost in bureaucracy. |
| 1. **Public Health**   **Education** | currently very little is done except for World Mental Health Day. |
| ***National public health education*** | Currently done voluntarily by various health workers and organisations at national and local levels. |
| ***National anti-stigma campaign*** | Mental health and mental illness stigmatised at all levels |
| ***Tackling stigma in health workers*** | Mental health patients and services stigmatised |
| ***Partnership between physical and mental health programmes*** | No partnership |
| ***Mental health education in schools*** | rarely happens |
|  |  |
| 1. **District Health Management Teams (DHMTs)** | In most of the districts, there is inadequate mental health representation on DHMTs.  mental health not on agenda of DHMTs  mental health rarely included in annual district plans |
| 1. **Traditional**   **healers** | Many people with mental illness consult traditional healers either exclusively or simultaneously. Practice is variable, some may do well, (eg some hysterical conversion syndromes, depression and anxiety) others are undoubtedly harmed or neglected. (eg epilepsy, psychosis) .  TBAs see many mothers with post natal depression and post natal psychosis  No liaison with traditional healers  little knowledge of efficacy and side effects of herbs |
| **Information**  **systems** | No comprehensive system of mental health information. |
|  | No mental health outcome indicator |
| 1. **Liaison with**   **Police** | Police help bring people to inpatient units but are not generally familiar with mental health act or good practice.  Key players would like closer collaboration  No systematic liaison at local level  Police commission is enthusiastic for collaboration |
|  | No education for police about mental illness. But Police trainers would like to know about mental illness. |
|  | Police are often unaware of what local services are available. |
|  | Police have no training on managing violent incidents in people with mental illness. |
|  | Police have no good practice guidelines on handling people with mental disorders. |
|  | Police have an important role in implementation of mental health act of 1989 |
|  | Police commission would like assistance with stress and mental health problems and alcohol abuse in police officers. |
|  | Police have no occupational health policy or service let alone occupational mental health. |
| 1. **Liaison with**   **prisons** | 89 prisons, 2 borstals and 1 youth corrective centre in Kenya. Prison population is 35000-40000. 1400 staff.  Both MoH and Prisons department would like closer links on mental health. |
|  | Need better information about mental health needs of prisoners, but anecdotally huge problem |
|  | No mental health in training of prison health workers |
|  | No guidelines about mental health for prison staff. |
|  | prison officers very stressed |
|  | some people have waited 18 years to go trial because npt well enough to go to court |
| **consistency of various legislation** | lack of harmonisation of various acts |
|  | Inadequate facilities to look after the mentally ill in prisons, There are many mentally ill prisoners who are not getting treatment because of long periods of waiting to go to Mathari hospital |
| **Forensic services** | centralised at Mathari Hospital, no provincial facilities |
| 1. **Liaison with**   **Health education** | Very little mental health is included in health education |
| 1. **Substance Abuse** |  |
| ***services*** | there are no special health services for drug abuse. Instead , cases are supported by the general health services or by psychiatric units. |
| ***NGOs for substance abuse*** | NACADA is co-ordinating national public awareness about substance abuse. SCAD is involved in creating awareness in schools and colleges. |
| **13. Ministry of Education, Science and Technology** |  |
| ***integration of mental health issues into work of Min of Ed*** | 30-40% of population under Min of Education. Children under many constraints affecting their education including poverty, nutritional status, sanitation, water supply, mental state of parents, lack of place for home work, lack of place for play |
| ***teacher awareness of mental health issues*** | teachers are not very aware of mental health and emotional issues |
| ***guidance and counselling*** | is provided in schools |
| ***health education for children*** | focuses on physical health and not on mental health |
| ***teacher awareness of occupational health and safety issues*** | teachers not taught about health and safety |
| ***teacher awareness about sources of assistance*** | teachers not aware of help available at PHC, district and provincial level, and frequently refer straight to Mathari which is rarely appropriate |
| **14.Ministry for Home Affairs, Children's Dept** |  |
|  | Only 1 children's officer per district |
|  | lack of knowledge about mental health issues in children |
|  | no inclusion of mental health issues in training of children's officers |
|  | lack of harmonisation and mutual awareness of legal frameworks |
| **15.Social welfare and rehabilitation** |  |
| ***awareness of mental health issues*** | Staff have no knowledge of mental health issues.  . |
| ***Link between Ministry of Health and Ministry of Social Services*** | No systematic collaboration at all at present |
| ***Detailed links at all levels.*** | ditto |
| ***skills for field officers*** | no exposure to mental health in training |
| ***literature for distribution to clients*** | none available |
| ***knowledge of social workers about referral pathways*** | not present |
| ***availability of occupational rehab at village level*** | not present, but Social Service s keen to collaborate on this by working through the village health committees |
| ***curriculum for social workers*** | Mental health not included |
| ***postgraduate course for social work*** | Mental health not included |
| ***training in key legislation*** | Not done |
| **16.Ministry of Labour** |  |
|  | lack of awareness and understanding of mental health issues in workplace |
|  | lack of Kenyan data on mental health in workplace, although now have some from Maseno study |
| ***health and safety committees in workplaces*** | A new law is coming to encourage workplaces to establish health and safety committees, one of whose tasks is to establoish health policies |
| ***health policies in workplaces*** | Not present |
| **17. NGOs** |  |
| ***Kenya Mental Health Association*** | Kenya Mental Health Association is non-functioning. |
| ***Kenya Schizophrenia Fellowship*** | Kenya Schizophrenia Fellowship has been very active, establishing assertive support for patients and families through advocacy, sharing experience and resources. |
| ***AMREF*** | AMREF has  -a CBR programme.in Kibwezi dealing with disability  -programme for street children in Dagoretti  -drug dependence and poverty reduction in kibera  -school health programmes dealing with counselling in schools  -Bomb blast programme-the medical assistance programme and disaster response unit includes psychological aspects-offer counselling, support groups and outreach. |
| ***Amani counselling centre*** | Provides counselling services for people with emotional problems and training for counsellors. services to Nairobi, Mombasa, Nyeri and kisumu. Diploma in collaboration with University of Cork, certificate level and short courses |
|  | FIDA provides services -for survivors of gender based violence,  -legal aid  -monitoring of trends in women human rights violations, violence, forced marriages, FGM  -gender and human rights awareness programmes in collaboration with police and medical services |
|  | KPA and KNA are active. |
| **18. Human resource development** |  |
| ***medical undergraduates*** | input into medical undergraduate training has improved |
| ***trainee psychiatrists*** | 2) Psychiatric training is sustainable but a bit in bred. |
| ***clinical psychologists*** | No clinical psychologists |
| ***nurses*** | Nurse numbers falling because of introduction of fees for training |
|  | Nurse training deficient in common mental disorders. |
| ***Occupational therapists*** | OT shortage because recruited by US and UK. |
| ***clinical officers*** | Clinical officers are deficient in mental health training. |
| ***social workers*** | No social workers, and no organised training for medial social workers |
| ***community (village) health workers*** | No mental health in training for community health workers |
| ***systematic continuing education*** | No regular systematic continuing education programmes for staff, much of which could be multidisciplinary. |
| ***postgraduate diploma*** | No postgraduate psychiatry diploma course for nurses, clinical officers etc |
| ***recruitment and retention*** | Difficulty recruiting and retaining staff outside Nairobi |
| **Kenya Medical Training College** | The KMTC trains all cadres of primary health care workers in Kenya. The majority of the courses have an inadequate mental health component. |
| ***training of trainers at PHC training centres*** | we have done Chulaimbo but not the others |
| ***College of Health Sciences, Department of Psychiatry*** | Department of psychiatry offers  -undergraduate training in psychiatry  -mental health to other disciplines  -Master of medicine in Psychiatry  Master of Science on Clinical Psychology |
| **19. Legislation** | Mental Health Act Chapter 2.48 of 1989 Laws of Kenya. |
|  | The rules and regulations for the new act have never been developed. |
|  | There has not yet been a systematic training programme for key staff in the new act. |
| **20.. Research and Development** | Very little. |
| **21. Refugees and displaced persons** | Many refugees in northern Kenya from Ethiopia. Some Congolese and Rwandans. |
| **22, other vulnerable groups** | eg women, children, physical and sensory disability, homeless |
