# Supplementary material for: Mental health policy in Kenya -an integrated approach to scaling up equitable care for poor populations
Source: Int J Ment Health Syst. 2010 Jun 28;4:19. doi: 10.1186/1752-4458-4-19 (PMC2907308; doi:10.1186/1752-4458-4-19)
Supplement: Additional file 2 — Kenya Mental Health Strategic Action Plan 2004 [file 1752-4458-4-19-S2.DOC]

**Additional file 2 - Kenya Mental Health Strategic Action Plan 2004**

.

| **Policy Component** | **Recommendations** |
| --- | --- |
| 1. **Position of**   **Mental Health in Ministry** |  |
| ***Director of Mental Health*** | Ensure Director of Mental Health MOH is able to devote full time rather than part time to post, by appointing separate Director of Mathari Hospital. |
| ***Board of Mental Health*** | Make adequate provision for KBMH to meet at least quarterly as stipulated in the Act. |
|  | Enhance effectiveness of Kenya Board of Mental Health |
|  | Ensure Board addresses the range of key issues needed for the implementation of the mental health policy |
| ***Mental health policy*** | Prepare mental health policy, vision statement, mission and strategic plan |
| ***linkage to PRSP and economic recovery plan*** | include mental health in PRSP |
| ***linkages to broad health and social policy*** | Raise the political profile of mental health and ensure the inclusion of mental health in all relevant policies |
| ***Support to Director of Mental Health*** | Support mental health division with more staff .  The Director of Mental Health to write staffing norms, jobs description and justification. |
| ***Prioritisation of mental health*** | Integrate Mental Health Strategic Plan into National Health Sector Strategic Plan |
| ***representation in donor meetings by Division of Mental Health*** | Ensure that when the Mental Health Strategic Plan is incorporated in the National Health Sector Strategic plan, there will be a Division of Mental Health representation in the donor meetings |
| ***Collaboration with Directorate of Preventive and Promotive Health services*** | Sensitise the heads of Divisions and Programme Managers on the role of mental health on HIV/AIDS, TB and leprosy, reproductive health, child health, PHC etc |
| ***Collaboration with Directorate of Policy and Planning*** | Collaborate with health planners within the ministry and liaise with ministry of national planning and economic development. |
| ***Collaboration with Human Resources*** | Enhance training of personnel in mental health by appointing Director of Mental Health a member of Ministerial Training Committee |
| ***mental health awareness of senior MOH officials*** | Enhance mental health awareness of DMS,PS, Deputy secretaries, finance dept heads, economists, treasury officials, division heads, PMS and MOHs |
| ***links with other key ministries eg criminal justice, home affairs, social welfare, education, labour.*** | Develop mechanism for overall inter-ministry linkages as well as joint work on specific issues eg mental health education in schools |
| 1. **Primary**   **Health Care** | Improve mental health component of basic training of clinical officers, primary care nurses, community health workers. |
| ***Continuing education (in- service training)*** | Inclusion of mental health in regular continuing education for PHC staff |
| ***Role of village health workers*** | Extend role of village health worker to cover community mental health education, detection of illness and referral, support between clinic appointments. |
|  | Inclusion of mental health in regular continuing education for village heath workers |
| ***Dialogue with traditional healers*** | Develop mental health training module for traditional healers.  Include traditional healers in CE sessions |
| ***Guidelines*** | Adapt, pilot and use WHO primary care guidelines for common mental disorders.. |
| ***PHC health information*** | information system in primary health care should include at least the 12 basic categories of mental illness. |
| ***essential medicines kit*** | Ensure adequate supply of essential medicines to PHC |
| ***transport for outreach*** | Consider bicycles/motor bikes as part of overall strengthening of PHC, as well as one vehicle per district |
| ***access to rehabilitation at PHC level*** | Main goal is for home -community based rehabilitation. Needs partnership between PHC, social services and NGOs to provide. |
| ***stigma at PHC level*** | stigma campaign-see public education below. |
|  |  |
| **Links between**  **primary and**  **secondary care** | Develop criteria for shared care, referral and mutual support. |
| ***referral pathways*** | MOH to strengthen integrated referral system |
| ***standards*** | MOH to develop quality of care guide for mental health care settings at all levels |
| ***transport for districts*** | District PHC transport matrix to be strengthened to support mental health supervision |
| ***communication*** | Phones, fax, email, stamps |
| ***regular supervision of PHCs by districts*** | Systematic supervision of PHC by districts needs to be built into district plans and budgets, and transport matrix. |
| ***training for districts in how to supervise and support PHCs*** | Districts need training in how to give such supervision to PHCs ie both in content and style of teaching/supervision |
| **4. Secondary care** |  |
| ***District, provincial and zonal and national tiers*** | Consider how provincial hospitals can best support their districts.  Institute regular supervision/support between tiers, especially provinces to districts |
| ***Inpatient units*** | ***Capital programme to build psychiatric ward in each district hospital***  of 10-20 beds for 100,000 population for short stay admissions for people who cant be cared for at home while acutely ill. |
| ***linkages between mental health service and physical health service*** | The psychiatric service needs to be treated as an integral part of the district hospital, with good liaison between staff, to address comorbidity and to ensure equity between mental and physical patients |
| ***outpatient clinics*** | Assess need to mental health OPD at district level, and work on staffing |
| ***Community outreach services*** | Develop manual on developing comprehensive district community services (inpatient units, outpatient clinics, community outreach, OT programmes, and community education). |
| ***Availability of medicines*** | Revise essential medicine list to include psychotropics, and order appropriately. |
| ***Good practice guidelines for secondary care*** | Develop specialist mental health guidelines. |
| ***Rehabilitation activities on inpatient wards*** | Manual on ward activities  Standards of care for hospitals settings |
| ***Rehabilitation facilities at district level*** | Develop occupational rehabilitation at district hospital level , and ideally at PHC level. Skills to facilitate home based rehabilitation to be emphasised. |
| ***availability of psychological treatments in secondary care*** | Seminars for health workers on community working, engagement with families prior to discharge, rehabilitation, psycho-education and addictions. |
| ***Delivery of support and supervision to PHC*** | District mental health teams to regularly visit all primary care centres to give support and supervision, and discuss liaison issues and ? give regular continuing education. |
| ***length of admissions*** | Assess social and occupational rehabilitation needs of long stay patients, and formulate active management plans with regular review. |
| ***national hospitals*** | Intensive revitalisation of Gilgil, Port Ritz, Kisumu and Mathari hospitals, with a view to including general health services within the package. |
| 1. **Public Health**   **Education** | Intensify Public Health education at all levels. |
| ***National public health education*** | Make more systematic at all levels. |
| ***National anti-stigma campaign*** | Develop campaign |
| ***Tackling stigma in health workers*** | Tackle stigma in health workers |
| ***Partnership between physical and mental health programmes*** | Make case that mental health helps achieve better physical health outcomes and vice versa. |
| ***Mental health education in schools*** | Include mental health in schools and school health programmes. |
|  | Develop mental health and child development modules in teacher training. |
| 1. **District Health Management Teams (DHMTs)** | As part of integrated services, there should be adequate mental health representation on DHMTs. |
|  | Mental health to be regular item on agenda to consider adequacy of supply of essential medicines, continuing education, support and co-ordination, transport for co-ordination and for outreach, seminars for personnel in dispensaries and health centres, liaison with police and prisons at local levels. |
|  | Mental health to be included in annual district plans |
|  | Mental health to be included in the national essential health package |
| 1. **Traditional**   **healers** | Therefore dialogue is important to encourage early referral where necessary and reduce harmful practices  Training programmes for TBAs should include mental health  Give diagnostic algorithms to encourage referral of serious cases e.g. malaria, epilepsy, psychosis, severe depression  assess scope for safe liaison  Research herbal medicines and other interventions |
| **Information**  **systems** | Integrated mental health information system between dispensaries, health centres and hospitals in order to assist in shared care, calculations of needs for care, needs for essential medicines and to support MoH in its planning functions. |
|  | Pilot HONOs as an outcome indicator at Isiolo and Meru as indicator districts. |
| 1. **Liaison with**   **Police** | Establish close liaison between mental health services and police |
|  | All decision-makers to come together in a joint national seminar to develop collaboration. |
|  | Joint meetings and seminars between mental health services and police at, provincial and district levels. |
|  | Include mental health in police training. |
|  | inform police officers of mental health facilities at district level. |
|  | Training re-handling and documenting violent incidents, especially in people with mental illness |
|  | Guidelines for police on mental disorders. |
|  | Rules and regulations currently taught at police training college are in accordance with MTA 1949 rather than MHA 1989 |
|  | Mental health input into police training school.  Give police staff in hardship postings more frequent leave and restrict to two years.  . |
|  | Occupational mental health policy and services for police. |
| 1. **Liaison with**   **prisons** | Closer links between MoH and prison department. |
|  | Mental health needs of prisoners to be researched. Prison service would like to do collaboratively with MOH |
|  | Mental health modules for basic training and continuing education of prison officers |
|  | Mental health modules for basic training and continuing education of prison health workers |
|  | Develop mental health guidelines for prisons. |
|  | Kenya Medical Association in *prisons to be extended to include mental health. |
|  | seminars for prison officers in stress management |
| ***court orders*** | court orders should direct mentally ill people directly to hospital rather than prisons |
|  | people should only stay in prison for 1 month to assess capability of standing trial. Then they should be orders to be in a mental hospital |
|  | Hospital medical superintendent should do quarterly reports on capability of standing trial |
| **consistency of various legislation** | Criminal Procedures Code , chapter 75 laws of Kenya, the Persons act chapter 90 Laws of Kenya and the Mental Health Act chapter 248 should be harmonised in relation to mentally disordered offenders. |
|  | review need |
|  |  |
| **Forensic services** | . make secure facilities available for mentally disordered offenders at local level. |
| 1. **Liaison with**   **Health education** | Strengthen mental health in health education programmes. |
| 1. **Substance Abuse** |  |
| ***services*** | strengthen substance abuse in training programmes for health staff |
| ***NGOs for substance abuse*** | NACADA is co-ordinating national public awareness about substance abuse. SCAD is involved in creating awareness in schools and colleges. |
| **13. Ministry of Education, Science and Technology** |  |
| ***integration of mental health issues into work of Min of Ed*** | enhance awareness of Min of Ed and teachers |
| ***teacher awareness of mental health issues*** | enhance awareness in teachers and field officers,. |
| ***guidance and counselling*** | consider what more needs to be done |
| ***health education for children*** | add mental health to health education, and integrate across the curriculum. Curriculum review |
| ***teacher awareness of occupational health and safety issues*** | occupational health and safety training for teachers |
| ***teacher awareness about sources of assistance*** | teachers not aware of help available at PHC, district and provincial level, and frequently refer straight to Mathari which is rarely appropriate |
| **14.Ministry for Home Affairs, Children's Dept** |  |
|  | need closer links and networking at ministry level and at district level |
|  | enhance awareness at ministry level and set plans |
|  | basic training of children's officers and continuing education |
|  | harmonisation of legislation and regulations |
| **15.Social welfare and rehabilitation** |  |
| ***awareness of mental health issues*** | Social services department is keen to collaborate and would like training for their staff in the various sections of culture, gender, services for vulnerable groups, youth programmes |
| ***Link between Ministry of Health and Ministry of Social Services*** | establish regular liaison at ministry level |
| ***Detailed links at all levels.*** | establish regular liaison at programme level |
| ***skills for field officers*** | pilot training for field officers |
|  |  |
| ***literature for distribution to clients*** | prepare leaflets |
| ***knowledge of social workers about referral pathways*** | prepare information pack for social workers |
| ***availability of occupational rehab at village level*** | prepare pilot site for this |
| ***training in key legislation*** | Training for officers on mental health and orientation to specific issues such as Children’s Act. |
| **16.Ministry of Labour** |  |
| ***health and safety committees in workplaces*** | enhance attention to mental health as well as physical health and substance abuse |
| ***health policies in workplaces*** | Encourage workplaces to develop mental health policies |
| **17. NGOs** |  |
| ***Kenya Mental Health Association*** | Revive KMHA |
| ***Kenya Schizophrenia Fellowship*** | Support KSF to expand. Consider Kenya Schizophrenia Foundation to start medicines project as in Tanzania |
| ***AMREF*** | AMREF would like to collaborate on  -stigma for those with physical disability and mental illness.  -drug dependence programmes amongst schoolchildren and colleges  -database of all organisations offering counselling services  -operational research providing baseline information on mental illness  -strengthening capacity of rapid response services  -training of PHC providers |
| ***Amani counselling centre*** | need database of organisations engaged in training of counsellors  need regulatory body to set standards, vet training institutions.  In case of disaster, poor coordination of different organisations |
|  | Collaborate with FIDA to reduce family violence and create awareness and include more mental health in their interventions. Add domestic violence and child abuse to primary care guidelines and continuing education of primary care and police. FIDA to link with SCAD for preventive work in schools on gender and domestic violence . |
|  | Support mental health division. |
| **18. Human resource development** | . |
| ***medical undergraduates*** | Increase coverage of common mental disorders in line with WHO PHC |
| ***trainee psychiatrists*** | Arrange external teachers and attachments and exchanges. |
| ***clinical psychologists*** | Start basic training of clinic psychologists |
| ***nurses*** | Develop Incentives in training in mental health for nurses including scholarships; fund nurse training. |
|  | Train nurses in common mental disorders. |
| ***Occupational therapists*** | Train more OTs. |
| ***clinical officers*** | Put mental health into clinical officers basic training curriculum. |
| ***social workers*** | Develop cadre of social workers and include mental health training for social workers. |
| ***community (village) health workers*** | Include mental health in training of community health workers. Train them to follow up chronic mental patients in the community, including keeping a register, ensuring drug compliance and regular follow up. |
| ***systematic continuing education*** | Strengthen continuing education for all staff. |
| ***postgraduate diploma*** | develop such a course at KMTC |
| ***recruitment and retention*** | Incentives for retention and deployment outside Nairobi. |
| **Kenya Medical Training College** | Strengthen mental health in basic training of all cadres  harmonisation of mental health components with specific courses |
| ***training of trainers at PHC training centres*** | Capacity building and staff development for implementation of curriculum |
| ***medical social work*** |  |
| ***postgraduate diploma course*** |  |
| ***College of Health Sciences, Department of Psychiatry*** | Senate has approved some further Postgraduate Diplomas, yet to start, on Psychotrauma, Management of Drug Abuse and Clinical Psychiatry |
| **19. Legislation** | Need to urgently review and revise act. |
|  | Develop rules and regulations act to endure smooth implementation |
|  | Needs more training at national, provincial and local levels across relevant sectors of health, social welfare, police, prisons, NGOs. |
| **20.. Research and Development** | Increase collaboration between MOH and research activities to improve services. |
|  | Train health workers in basic research. |
|  | Epidemiology – prevalence of mental disorders at each level in system. |
|  | Health outcomes |
|  | traditional herbs, traditional interventions, and safe methods of liaison |
|  | mental health in workplace-size of problem and evaluation of interventions |
| **21. Refugees and displaced persons** | Need for mental health component in provisions of health services to refugee population, to include training of personnel and to link with UNHCR on mental health issues and services. |
